# Imaging-guided intervention of foot and ankle disorders

**DOI:** 10.1186/1757-1146-4-S1-I4

**Published:** 2011-05-20

**Authors:** George Koulouris

**Affiliations:** 1 Melbourne Radiology Clinic, East Melbourne, VIC 3002, Australia

## 

Numerous degenerative, traumatic, overuse and inflammatory conditions occur about the foot and ankle both specific to the region and also as part of generalised systemic processes. The risks, benefits, indications and contraindications of common imaging guided procedures will be discussed, such as the use of corticosteroids, autologous blood, platelet rich plasma, autologous tenocytes, alcohol, radiofrequency ablation and polidocanol, to name a few. The role that these interventions perform in the treatment algorithm will also be reviewed.

